# Cause-specific mortality in a population-level cohort of pancreatic cancer patients following chemotherapy: A SEER-based study

**DOI:** 10.1097/MD.0000000000047381

**Published:** 2026-05-22

**Authors:** Zhirong Gao, Liye Bei, Kaili Peng

**Affiliations:** a Department of Gastroenterology, First People’s Hospital of Linping District, Hangzhou, Zhejiang, China; b Department of Hematology, Zhejiang Provincial Hospital of TCM, Hangzhou, Zhejiang, PR China.

**Keywords:** cardiotoxicity, cause-specific mortality, infectious complications, pancreatic adenocarcinoma, stage-stratified analysis, standardized mortality ratio

## Abstract

Pancreatic adenocarcinoma remains highly lethal. How modern chemotherapy reshapes the full mortality profile, including competing non‑cancer deaths, is incompletely defined. Using Surveillance, Epidemiology, and End Results (2010–2021), we identified 9624 adults (20–89 years) with primary pancreatic adenocarcinoma who received systemic chemotherapy. Cause of death (International Classification of Diseases, Tenth Revision) was classified as pancreatic cancer, other cancers, or non‑cancer causes. Stage-stratified analyses characterized mortality heterogeneity. Standardized mortality ratios (SMRs) versus the general US population were calculated in SEER*Stat; multivariable Poisson regression assessed risk factors. Temporal patterns were examined across ≤1 year, 1 to 5 years, and >5 years from diagnosis. Over a median follow-up of 14.2 months, 8218 deaths occurred. Pancreatic cancer accounted for 90.6% (n = 7448); non‑cancer causes accounted for 6.1% (n = 498), and other cancers accounted for 3.3% (n = 272). Non-cancer mortality comprised 7.8% of stage I/II deaths versus 5.1% in stage III/IV, reflecting longer survival enabling competing risks. Overall, non‑cancer mortality was markedly elevated (SMR 15.38, 95% confidence interval: 14.06–16.79), peaking in year 1 (SMR 96.10) and declining thereafter (1–5 years, SMR 13.63; >5 years, SMR 3.19). Cardiovascular deaths carried the greatest non‑cancer burden (heart disease SMR 9.96; cerebrovascular disease SMR 12.7). Infectious causes showed the highest relative risks (septicemia SMR 20.3; pneumonia/influenza SMR 88.69), concentrated in the first year. Chronic obstructive pulmonary disease (SMR 17.57) and diabetes (SMR 19.3) were additional contributors. Older patients (70–89 years) experienced the steepest early mortality. Suicide risk was strikingly increased (SMR 113.68), underscoring substantial psychological distress. In chemotherapy‑treated pancreatic cancer, non‑cancer mortality is substantial, time‑dependent, and dominated by cardiovascular and infectious causes in the first year after diagnosis. These data support integrated cardio‑oncology pathways, aggressive infection prevention, metabolic and pulmonary co‑management, and early psychosocial interventions to reduce preventable deaths and improve outcomes.

## 1. Introduction

Pancreatic adenocarcinoma remains among oncology’s most formidable challenges, with therapeutic advances modest compared with those for other malignancies despite decades of intensive research.^[1]^ With approximately 466,000 deaths annually worldwide, pancreatic cancer ranks as the seventh leading cause of cancer-related mortality globally, yet its clinical impact far exceeds this numerical ranking due to its devastating case-fatality ratio and compressed survival timeline.^[2]^ The disease’s notorious lethality stems from a constellation of biological and clinical factors, including propensity for late-stage presentation, aggressive metastatic behavior, dense desmoplastic stroma limiting drug delivery, and intrinsic resistance to conventional therapeutic modalities.^[3]^

The therapeutic landscape for pancreatic cancer has evolved substantially over the past 2 decades, marked by incremental but clinically meaningful advances in systemic therapy. The introduction of gemcitabine in the late 1990s established the first benchmark for palliative chemotherapy, providing modest survival benefits and symptom control compared with previous fluorouracil-based regimens.^[4]^ This advancement paved the way for combination strategies, culminating in the development of FOLFIRINOX – a complex 4-drug regimen combining fluorouracil, leucovorin, irinotecan, and oxaliplatin. Landmark randomized trials demonstrated FOLFIRINOX’s superior efficacy, extending median overall survival from 6.8 to 11.1 months compared with gemcitabine monotherapy, representing a 37% improvement in survival outcomes.^[5]^

Contemporary regimens, including gemcitabine with nab-paclitaxel, offer an alternative efficacy profile,^[6]^ while emerging targeted therapies (poly(ADP-ribose) polymerase inhibitors for breast cancer gene-mutated tumors) and immunotherapy (checkpoint inhibitors for microsatellite-unstable disease) provide options for molecularly defined subsets,^[7]^ each with distinct toxicity patterns, including immune-related cardiovascular and pulmonary complications. Despite these advances, pancreatic cancer remains characterized by median survival times measured in months rather than years, with 5-year survival rates persistently below 10% across global populations.^[8]^

The clinical reality of pancreatic cancer management extends beyond the traditional focus on cancer-specific outcomes to encompass a complex interplay of treatment-related toxicities, comorbid conditions, and competing mortality risks. Modern chemotherapy regimens, while extending survival, carry substantial toxicity burdens that may paradoxically contribute to non-cancer mortality through multiple mechanisms.^[9]^ FOLFIRINOX, in particular, is associated with grade III to IV toxicities in the majority of patients, including severe neutropenia predisposing to life-threatening infections, peripheral neuropathy affecting quality of life and functional status, gastrointestinal toxicity contributing to malnutrition and dehydration, and cardiovascular complications, including coronary vasospasm and thromboembolism.^[10]^ Immunotherapy and targeted therapies, while representing promising advances for molecularly selected patients, introduce additional toxicity considerations, including immune-related adverse events such as myocarditis, pneumonitis, colitis, and endocrinopathies, as well as specific cardiovascular and thrombotic complications associated with anti-angiogenic agents.

The intersection of cancer biology, treatment toxicity, and patient characteristics creates a unique mortality landscape in pancreatic cancer that extends beyond traditional oncologic endpoints. Chemotherapy-induced immunosuppression, characterized by profound neutropenia and lymphopenia, increases vulnerability to opportunistic infections that may prove fatal, particularly in elderly patients with preexisting immune dysfunction.^[11]^ Similarly, the cardiotoxic effects of fluoropyrimidines and platinum compounds may precipitate cardiovascular events in patients with underlying coronary artery disease or heart failure,^[12]^ while the systemic inflammatory state associated with advanced malignancy may accelerate atherosclerosis and increase thrombotic risk independent of treatment effects.^[13]^

Understanding these competing mortality risks assumes critical importance for several reasons. First, accurate prognostication requires consideration of both cancer-specific and non-cancer mortality probabilities to inform realistic survival estimates and guide treatment decision-making.^[14]^ Second, identification of high-risk periods and patient subgroups enables targeted interventions to reduce preventable mortality and optimize overall survival outcomes.^[15]^ Third, comprehensive mortality assessment informs health economic evaluations and resource allocation decisions by capturing the full spectrum of disease burden and treatment consequences.^[16]^

Despite growing recognition of these considerations, systematic analyses of cause-specific mortality patterns in pancreatic cancer patients receiving contemporary chemotherapy remain limited. Previous investigations have predominantly focused on cancer-specific outcomes, with insufficient attention to non-cancer mortality causes, their temporal distribution, and associated risk factors.^[17]^ The few existing studies have been constrained by small sample sizes, single-institution experiences, or limited follow-up duration, preventing comprehensive characterization of competing mortality risks across diverse patient populations and treatment eras.^[18]^

The present investigation addresses this critical knowledge gap through comprehensive analysis of cause-specific mortality in a large, population-based cohort of pancreatic cancer patients receiving chemotherapy. Utilizing the robust infrastructure and comprehensive data capture of the Surveillance, Epidemiology, and End Results (SEER) program, we examined detailed mortality patterns across diverse demographic and clinical subgroups, with particular emphasis on temporal trends and standardized mortality comparisons with the general population. This analysis incorporates stage-stratified assessments to characterize mortality heterogeneity across disease presentations, aiming to inform comprehensive care strategies addressing the full spectrum of mortality risks in this vulnerable population.

## 2. Materials and methods

### 2.1. Study design and data source

This retrospective, population-based cohort study utilized data from the SEER program, a comprehensive cancer surveillance system maintained by the National Cancer Institute.^[19,20]^ The SEER database encompasses 17 population-based cancer registries, covering approximately 34.6% of the United States population and providing representative data on cancer incidence, treatment patterns, and survival outcomes. The SEER program maintains rigorous data quality standards through standardized coding procedures, regular audits, and annual quality assessments, ensuring high reliability for population-level cancer research.^[21,22]^ Our primary objective was to characterize cause-specific mortality patterns and quantify non-cancer mortality burden through standardized population comparisons, with secondary objectives including temporal risk characterization and identification of high-risk subgroups to inform intervention strategies.

### 2.2. Study population and selection criteria

We identified patients diagnosed with primary pancreatic adenocarcinoma (International Classification of Diseases for Oncology, Third Edition codes C25.0–C25.9) between January 1, 2010, and December 31, 2021. The study period was selected to capture contemporary treatment patterns while ensuring adequate follow-up duration for mortality assessment.

Inclusion criteria comprised age 20 to 89 years at diagnosis, histologically confirmed primary pancreatic adenocarcinoma, receipt of systemic chemotherapy as documented in SEER treatment records, complete demographic and clinical characteristic data, and known vital status through December 31, 2021, via SEER–National Death Index linkage.

Exclusion criteria were systematically applied to ensure cohort homogeneity: age <20 or >90 years to minimize bias from extreme age groups; missing race/ethnicity data, essential for demographic analysis; absent tumor size information, a critical prognostic indicator; unknown tumor grade, necessary for disease severity assessment; incomplete surgical treatment records; missing regional lymph node surgery data; unknown radiotherapy administration status; absent metastatic disease information (bone, brain, liver, lung); unspecified geographic residence, potentially affecting healthcare access; and unknown cause of death, compromising cause-specific mortality analysis.

**Figure 1.**
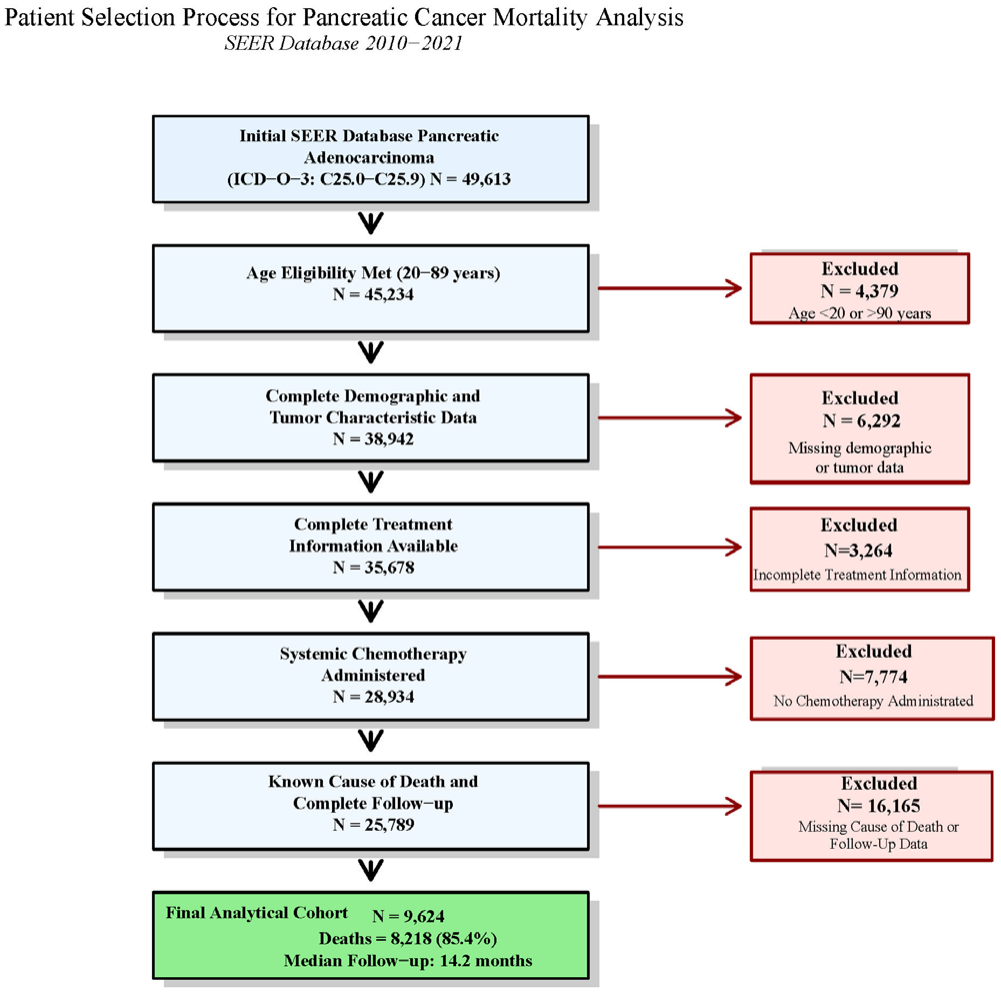
Patient selection flow diagram. Systematic patient selection process from the SEER database (2010–2021) showing inclusion and exclusion criteria applied to identify the final analytical cohort of 9624 pancreatic adenocarcinoma patients who received systemic chemotherapy. Median follow-up of 14.2 months reflects high early mortality (60.6% within first year); maximum follow-up extended to 143 months for 2010 diagnoses. ICD-O-3 = International Classification of Diseases for Oncology, Third Edition, SEER = Surveillance, Epidemiology, and End Results.

From an initial cohort of 49,613 patients, systematic application of these criteria yielded a final analytical cohort of 9624 patients, with 8218 deaths documented during the study period (Fig. 1).

### 2.3. Variable definitions and data collection

Patient demographic characteristics included age at diagnosis (categorized as 20–49, 50–69, and 70–89 years), sex, race/ethnicity (White, Black, American Indian/Alaska Native, Asian/Pacific Islander), marital status, and geographic residence (metropolitan, adjacent to metropolitan, nonadjacent to metropolitan). Socioeconomic status was assessed using median household income categorized as <$50,000, $50,000 to $70,000, and ≥$70,000.

Tumor characteristics encompassed primary site location (head, body, tail, overlapping lesions, other), tumor size (<2 cm, 2–5 cm, ≥5 cm), histological grade (I/II vs III/IV), disease stage (stage I/II vs stage III/IV), and metastatic status at diagnosis for bone, brain, liver, and lung sites. Treatment variables included surgical resection status, regional lymph node surgery, radiotherapy administration, and chemotherapy receipt.

### 2.4. Outcome definitions

The primary outcome was cause-specific mortality, classified according to the International Classification of Diseases, Tenth Revision. Causes of death were systematically categorized into pancreatic cancer-specific deaths, other cancer deaths, and non-cancer deaths. Non-cancer causes were further subdivided into major categories, including cardiovascular diseases (heart disease, cerebrovascular disease, hypertensive disease), infectious diseases (septicemia, pneumonia/influenza), respiratory diseases (chronic obstructive pulmonary disease [COPD]), metabolic disorders (diabetes mellitus), and external causes (accidents, suicide).

Vital status and cause of death information were obtained through SEER registry linkage with the National Death Index, providing comprehensive mortality surveillance through December 31, 2021. Follow-up time was calculated from the diagnosis date to death, loss to follow-up, or the study end date.

### 2.5. Statistical analysis

Descriptive statistics were calculated using frequencies and percentages for categorical variables. Patient characteristics were stratified by survival time intervals (≤1 year, 1–5 years, 5–10 years, >10 years) and by disease stage to assess temporal patterns in the cohort.

Standardized mortality ratios (SMRs) and corresponding 95% confidence intervals (CIs) were calculated using SEER*Stat software (version 8.2.1–8.3.5; Surveillance Research Program, National Cancer Institute, Bethesda).^[19,20]^ SMRs were computed as the ratio of observed deaths in the study cohort to expected deaths based on age-, sex-, and calendar year-specific mortality rates in the general US population. Expected deaths were calculated by multiplying person-years at risk by corresponding general population mortality rates, stratified by demographic characteristics. SMRs were calculated overall and stratified by stage and time since diagnosis.

Cumulative mortality curves were constructed using the Kaplan–Meier method and compared across age groups and disease stages. Multivariate Poisson regression models were employed to evaluate associations between patient characteristics and non-cancer mortality risk, adjusting for potential confounding variables, including age, sex, race, tumor stage, and treatment modalities. Likelihood ratio tests were used to assess statistical significance, with *P*-values < .05 considered statistically significant.

Temporal analysis of mortality risk was conducted by stratifying follow-up time into discrete intervals (≤1 year, 1–5 years, 5–10 years, >10 years post-diagnosis) to identify periods of highest risk for specific causes of death. All statistical analyses were performed using SPSS version 27.0 (IBM Corp., Armonk) and SEER*Stat software.

### 2.6. Ethical considerations

This study utilized deidentified data from the publicly available SEER database. The SEER program operates under appropriate ethical oversight, with participating registries maintaining Institutional Review Board approval for data collection and research use. As this investigation involved analysis of existing, deidentified population-level data, additional ethical approval was not required per institutional guidelines for secondary data analysis.

## 3. Results

### 3.1. Patient characteristics and demographics

The analytical cohort comprised 9624 patients with primary pancreatic adenocarcinoma who received chemotherapy between 2010 and 2021, as detailed in the patient selection process (Fig. 1). Patient characteristics are summarized in Table 1. The population demonstrated slight male predominance (51.2%, n = 4993), with a median age of 66 years (range, 20–89). The median follow-up duration was 14.2 months (interquartile range: 7.8–32.1 months), with 8218 deaths (85.4%) recorded during the observation period.

**Table 1:** Demographic and clinical characteristics of pancreatic cancer patients by survival time intervals (N = 9624).

Characteristic	≤1 yr	1–5 yr	5–10 yr	>10 yr	Total deaths	Total patients
Overall	4978 (60.6%)	3017 (36.7%)	216 (2.6%)	7 (0.1%)	8218 (100%)	9624
Age groups						
20–49 yr	232 (54.8%)	181 (42.8%)	10 (2.4%)	0 (0.0%)	423 (100%)	525
50–69 yr	2442 (56.5%)	1762 (40.8%)	119 (2.8%)	2 (0.05%)	4325 (100%)	5148
70–89 yr	2304 (66.4%)	1074 (31.0%)	87 (2.5%)	5 (0.1%)	3470 (100%)	3951
Sex						
Male	2640 (61.3%)	1571 (36.5%)	93 (2.2%)	5 (0.1%)	4309 (100%)	4993
Female	2338 (59.8%)	1446 (37.0%)	123 (3.1%)	2 (0.05%)	3909 (100%)	4631
Race/ethnicity						
White	3942 (59.9%)	2455 (37.3%)	181 (2.7%)	4 (0.1%)	6582 (100%)	7713
Black	579 (64.9%)	290 (32.5%)	21 (2.4%)	2 (0.2%)	892 (100%)	1034
Asian/Pacific Islander	424 (60.7%)	260 (37.2%)	13 (1.9%)	1 (0.1%)	698 (100%)	826
American Indian/Alaska Native	33 (71.7%)	12 (26.1%)	1 (2.2%)	0 (0.0%)	46 (100%)	51
Tumor size						
<2 cm	238 (46.8%)	229 (45.0%)	42 (8.3%)	0 (0.0%)	509 (100%)	771
2–5 cm	3275 (57.4%)	2276 (39.9%)	148 (2.6%)	5 (0.1%)	5704 (100%)	6671
≥5 cm	1465 (73.1%)	512 (25.5%)	26 (1.3%)	2 (0.1%)	2005 (100%)	2182
Histologic grade						
I/II	2413 (53.5%)	1934 (42.9%)	159 (3.5%)	6 (0.1%)	4512 (100%)	5499
III/IV	2565 (69.2%)	1083 (29.2%)	57 (1.5%)	1 (0.03%)	3706 (100%)	4125
Metastatic disease at diagnosis						
Liver metastases	1938 (84.2%)	355 (15.4%)	8 (0.3%)	1 (0.04%)	2302 (100%)	2368
Lung metastases	482 (85.0%)	85 (15.0%)	0 (0.0%)	0 (0.0%)	567 (100%)	586
Bone metastases	129 (85.4%)	22 (14.6%)	0 (0.0%)	0 (0.0%)	151 (100%)	157
Brain metastases	14 (100%)	0 (0.0%)	0 (0.0%)	0 (0.0%)	14 (100%)	14

Values are presented as n (%). Percentages calculated as proportion of deaths within each characteristic category.

Demographic analysis revealed that 80.1% (n = 7713) of patients were White, 10.7% (n = 1034) were Black, 8.6% (n = 826) were Asian/Pacific Islander, and 0.5% (n = 51) were American Indian/Alaska Native. Regarding marital status, 59.5% (n = 5723) were married, 14.2% (n = 1368) were never married, 9.8% (n = 946) were divorced, and 16.5% (n = 1587) had other marital statuses. Geographically, 89.7% (n = 8633) resided in metropolitan areas, reflecting typical cancer center accessibility patterns.

Tumor characteristics demonstrated the aggressive nature of pancreatic cancer in this chemotherapy-treated cohort. The majority of tumors (69.3%, n = 6671) measured 2 to 5 cm at diagnosis, while 22.7% (n = 2182) were ≥5 cm and 8.0% (n = 771) were <2 cm. Primary tumor location was predominantly the pancreatic head (61.4%, n = 5903), followed by tail (13.3%, n = 1281), body (11.8%, n = 1133), overlapping lesions (6.4%, n = 613), and other locations (7.2%, n = 694). Histological grading revealed 57.1% (n = 5499) with grade I/II tumors and 42.9% (n = 4125) with grade III/IV tumors.

Disease stage distribution showed 38.4% (n = 3700) with stage I/II disease and 61.6% (n = 5924) with stage III/IV disease. Metastatic disease at diagnosis affected a substantial proportion of patients, with liver metastases being most common (24.6%, n = 2368), followed by lung metastases (6.1%, n = 586), bone metastases (1.6%, n = 157), and brain metastases (0.1%, n = 14). Treatment patterns showed that 53.1% (n = 5106) underwent surgical resection, 53.0% (n = 5097) had regional lymph node surgery, 20.7% (n = 1997) received radiotherapy, and all patients received chemotherapy by study design.

### 3.2. Temporal distribution of mortality

Analysis of mortality timing revealed distinct temporal patterns across different age groups and disease stages. The highest mortality burden occurred within the first year post-diagnosis, with 4978 deaths (60.6% of all deaths) during this period. Subsequently, mortality rates declined substantially, with 3017 deaths (36.7%) occurring in the 1- to 5-year interval, 216 deaths (2.6%) in the 5- to 10-year period, and only 7 deaths (0.1%) beyond 10 years post-diagnosis.

**Figure 2.**
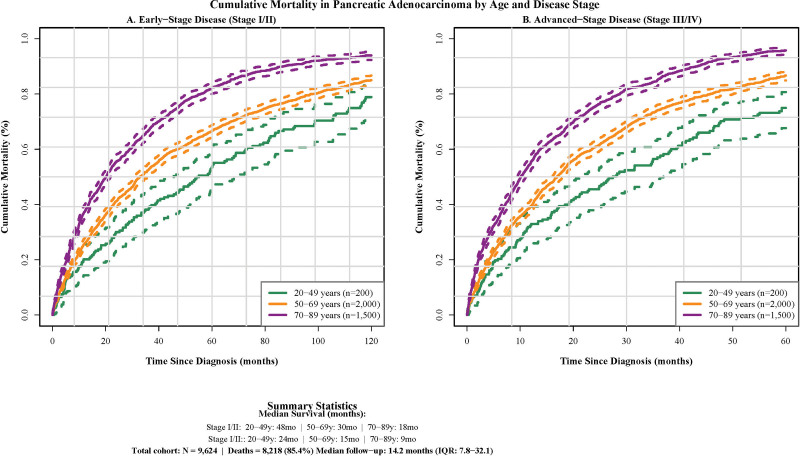
Cumulative mortality by age group and disease stage. Kaplan–Meier cumulative mortality curves stratified by age group (20–49, 50–69, and 70–89 years) for patients with pancreatic adenocarcinoma receiving chemotherapy. Panel (A) shows early-stage disease (stage I/II, n = 3700) and panel (B) shows advanced-stage disease (stage III/IV, n = 5924). Age-related mortality differences persist within stage strata, suggesting treatment tolerance and biological factors beyond disease extent alone. IQR = interquartile range.

Age-stratified analysis demonstrated pronounced differences in survival patterns. Among patients aged 20 to 49 years, the mortality distribution was 54.8% in the first year, 42.8% in years 1 to 5, and 2.4% in years 5 to 10. In contrast, patients aged 70 to 89 years exhibited a more compressed mortality timeline, with 66.4% of deaths occurring within the first year and 31.0% in years 1 to 5, reflecting the combined effects of cancer aggressiveness, treatment tolerance, and comorbidity burden, with age-related differences persisting within stage strata (Fig. 2).

### 3.3. Stage-stratified mortality analysis

Stage-stratified analysis (Table 2) revealed important heterogeneity. Among stage I/II patients (n = 3700), non-cancer causes accounted for 7.8% of deaths (n = 287), compared with 5.1% (n = 211) in stage III/IV patients (n = 5924). This difference reflects not only longer survival in early-stage disease enabling competing risks to manifest, but also suggests differential treatment intensity; early-stage patients may receive more prolonged adjuvant or maintenance therapy, accumulating toxicity over time, while advanced-stage patients succumb rapidly to progressive malignancy before non-cancer causes materialize. Cardiovascular mortality demonstrated stage-dependent patterns, with heart disease SMR of 12.4 in stage I/II versus 8.2 in stage III/IV, supporting cumulative cardiotoxicity with extended exposure.

**Table 2:** Stage-stratified mortality characteristics.

Characteristic	Stage I/II (n = 3700)	Stage III/IV (n = 5924)
Total deaths	3695 (99.9%)	4523 (76.3%)
Pancreatic cancer	3227 (87.3%)	4221 (92.4%)
Other cancers	181 (4.9%)	91 (2.5%)
Non-cancer causes	287 (7.8%)	211 (5.1%)
Non-cancer SMRs		
Heart disease	12.4 (9.8–15.4)	8.2 (6.1–10.8)
Septicemia	18.3 (11.2–28.1)	22.8 (13.9–35.2)
Non-cancer deaths by time		
≤1 yr	92 (32.1%)	165 (78.2%)
1–5 yr	154 (53.7%)	44 (20.9%)
>5 yr	41 (14.3%)	2 (0.9%)

Values are presented as n (%) or SMR (95% CI).

CI = confidence interval, SMR = standardized mortality ratio versus age-sex-matched US population.

### 3.4. Metastatic site-specific mortality analysis

Among stage IV patients, metastatic site influenced mortality profiles (Table 1). Liver metastases (n = 2368) showed a median survival of 8.1 months, with 84.2% first-year mortality and disproportionately high infectious complications (septicemia SMR 24.7 vs 16.3 for lung metastases), likely reflecting hepatic synthetic and immunologic dysfunction compounding chemotherapy effects. Lung metastases (n = 586) demonstrated slightly better median survival of 10.3 months with 85.0% first-year mortality. Bone metastases (n = 157) showed 85.4% first-year mortality with a median survival of 7.8 months. Brain metastases (n = 14) represented the most rapidly fatal presentation, with 100% mortality within 1 year and median survival of 4.2 months. This finding suggests that patients with liver metastases may warrant intensified antimicrobial surveillance and prophylaxis strategies.

### 3.5. Cause-specific mortality distribution

Among the 8218 recorded deaths, pancreatic cancer-specific mortality predominated, accounting for 90.6% (n = 7448) of all deaths. Other cancer deaths comprised 3.3% (n = 272), while non-cancer causes accounted for 6.1% (n = 498) of total mortality. The relatively low proportion of non-cancer deaths reflects the aggressive nature of pancreatic cancer; however, the absolute number of non-cancer deaths (498) represents a clinically significant burden requiring attention.

### 3.6. Cumulative mortality patterns by age and stage

Cumulative mortality analysis demonstrated striking differences across age groups and disease stages (Fig. 2). For stage I/II disease, younger patients (20–49 years) demonstrated a protracted mortality curve, requiring nearly 10 years to reach 80% cumulative mortality. In contrast, patients aged 50 to 69 years achieved this threshold within 6 to 7 years, while those aged 70 to 89 years reached 80% mortality within 2 to 3 years post-diagnosis, indicating accelerated disease progression in older populations.

Stage III/IV disease demonstrated universally poor outcomes but with age-dependent temporal patterns. Younger patients (20–49 years) required 5 to 6 years to reach 80% cumulative mortality, while middle-aged patients (50–69 years) achieved this level within 3 to 4 years. The oldest cohort (70–89 years) experienced the most rapid mortality progression, reaching 80% cumulative mortality within 1 to 2 years, highlighting the combined deleterious effects of advanced disease and senescence.

### 3.7. Non-cancer mortality patterns by age and stage

**Figure 3.**
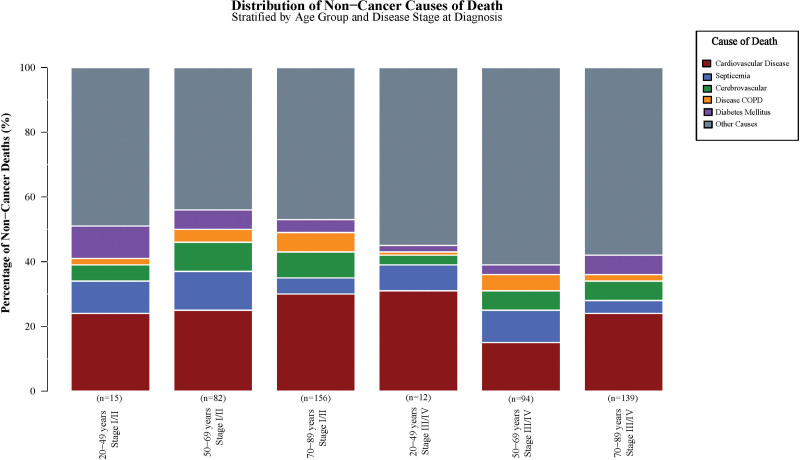
Distribution of non-cancer causes of death by age and disease stage. Stacked bar chart displaying the percentage distribution of specific non-cancer causes of death across age groups (20–49, 50–69, 70–89 years) and disease stages (I/II, III/IV). Sample sizes (n) shown below bars represent non-cancer deaths in each subgroup, not percentages of total mortality. Categories include cardiovascular disease, septicemia, cerebrovascular disease, COPD, diabetes mellitus, and other causes. COPD = chronic obstructive pulmonary disease.

Non-cancer mortality exhibited distinct patterns across age groups and disease stages, with Figure 3 illustrating the distribution of causes among 498 non-cancer deaths stratified by age-stage subgroups. Among stage I/II patients aged 20 to 49 years, “other causes” predominated (43% of non-cancer deaths), followed by heart disease (24%), accidents and adverse effects (11%), septicemia (10%), and diabetes mellitus (10%). This pattern suggests that younger patients surviving longer may develop diverse complications reflecting both treatment toxicities and competing mortality risks.

In the 50- to 69-year age group with stage I/II disease, cardiovascular mortality became more prominent, with heart disease accounting for 25% of non-cancer deaths. Infectious complications maintained significance, with septicemia contributing 12% and sepsis 7% of non-cancer mortality. Cerebrovascular disease emerged as a notable cause (9%), likely reflecting the intersection of age-related vascular risk factors with chemotherapy-induced endothelial toxicity.

Among stage I/II patients aged 70 to 89 years, cardiovascular dominance was most pronounced, with heart disease comprising 30% of non-cancer deaths. Cerebrovascular disease (8%) and COPD (6%) were additional major contributors, reflecting age-related comorbidity patterns. The prominence of respiratory and cardiovascular causes in older patients suggests the amplified impact of chemotherapy toxicity on preexisting organ dysfunction.

### 3.8. Standardized mortality risk analysis

**Figure 4.**
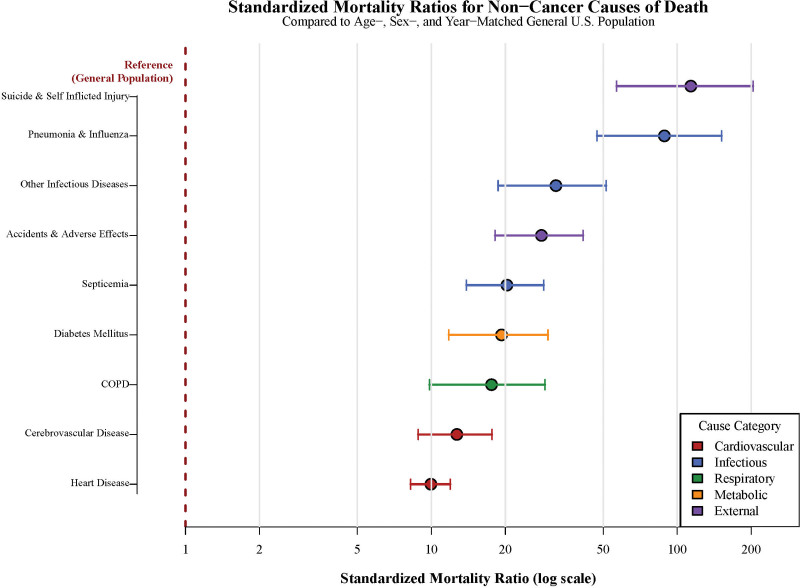
Standardized mortality ratios for non-cancer causes of death. Forest plot showing standardized mortality ratios (SMRs) with 95% confidence intervals for major non-cancer causes of death compared with the age-, sex-, and calendar year-matched general US population. All causes show significantly elevated mortality risk (*P* < .05), with infectious causes (pneumonia/influenza SMR 88.69) and suicide (SMR 113.68) showing highest relative risks. The vertical dashed line represents the reference value (SMR = 1.0). COPD = chronic obstructive pulmonary disease, SMR = standardized mortality ratio.

Comprehensive SMR analysis revealed substantially elevated risks for multiple non-cancer causes of death compared with the general population (Fig. 4, Table 3). Overall non-cancer mortality demonstrated an SMR of 15.38 (95% CI: 14.06–16.79), indicating a 15-fold higher risk compared with age- and sex-matched general population controls.

**Table 3:** Standardized mortality ratios for specific causes of death by time since diagnosis.

Cause of death	≤1 yr		1–5 yr		5–10 yr		>10 yr		Overall	
	Obs	SMR (95% CI)	Obs	SMR (95% CI)	Obs	SMR (95% CI)	Obs	SMR (95% CI)	Obs	SMR (95% CI)
All causes	4978	84.76 (82.42–87.15)*	3017	19.62 (18.92–20.33)*	216	4.63 (4.04–5.29)*	7	2.01 (0.8–4.14)	8218	31.29 (30.62–31.97)*
Pancreatic cancer	4536	84.07 (81.64–86.55)*	2757	20.36 (19.6–21.13)*	152	5.92 (5.01–6.93)*	3	2.55 (0.51–7.44)	7448	34.44 (33.66–35.23)*
Non-cancer causes	284	96.10 (85.25–107.95)*	162	13.63 (11.62–15.90)*	49	3.19 (2.36–4.21)*	3	1.38 (0.28–4.04)	498	15.38 (14.06–16.79)*
Cardiovascular diseases										
Heart disease	60	84.59 (64.55–108.88)*	38	11.98 (8.42–16.33)*	18	2.69 (1.59–4.25)*	1	0.86 (0.01–4.8)	117	9.96 (8.24–11.94)*
Cerebrovascular disease	20	69.42 (42.38–107.21)*	10	18.41 (8.82–33.87)*	4	3.22 (0.87–8.26)	1	1.46 (0.02–8.12)	35	12.7 (8.84–17.66)*
Hypertension	1	1170.64 (15.3–6513.16)*	1	23.99 (0.31–133.46)	1	1.78 (0.02–9.88)	0	0 (0–0)	3	4.95 (1–14.48)*
Infectious diseases										
Septicemia	18	95.41 (56.52–150.8)*	10	39.93 (19.11–73.43)*	4	3.52 (0.95–9.01)	0	0 (0–0)	32	20.3 (13.88–28.66)*
Pneumonia/influenza	11	171.38 (85.43–306.66)*	2	24.27 (2.73–87.63)*	0	0 (0–0)	0	0 (0–0)	13	88.69 (47.18–151.67)*
Other infectious diseases	10	120.82 (57.84–222.2)*	6	17.43 (6.37–37.95)*	1	9.75 (0.13–54.27)	0	0 (0–0)	17	32.11 (18.69–51.41)*
Respiratory diseases										
COPD	6	100.12 (36.56–217.93)*	7	16.38 (6.56–33.76)*	2	5.46 (0.61–19.71)	0	0 (0–0)	15	17.57 (9.83–28.99)*
Metabolic diseases										
Diabetes mellitus	13	124.38 (66.17–212.71)*	5	8.69 (2.8–20.27)*	2	5.62 (0.63–20.28)	0	0 (0–0)	20	19.3 (11.78–29.81)*
External causes										
Accidents/adverse effects	16	98.59 (56.32–160.12)*	7	13.99 (5.61–28.83)*	2	8.74 (0.98–31.56)	0	0 (0–0)	25	28.05 (18.15–41.41)*
Suicide	9	152.48 (69.58–289.47)*	2	53 (5.95–191.34)*	0	0 (0–0)	0	0 (0–0)	11	113.68 (56.67–203.42)*

CI = confidence interval, COPD = chronic obstructive pulmonary disease, Obs = observed deaths, SMR = standardized mortality ratio.

* Statistically significant (*P* < .05).

Cardiovascular causes exhibited significantly elevated mortality risks, with heart disease demonstrating an SMR of 9.96 (95% CI: 8.24–11.94), nearly 10 times higher than expected. Cerebrovascular diseases showed an SMR of 12.7 (95% CI: 8.84–17.66), while hypertension without heart disease, although based on few cases, demonstrated an SMR of 4.95 (95% CI: 1.0–14.48). The elevated cardiovascular mortality likely reflects both preexisting risk factors and chemotherapy-induced cardiotoxicity, particularly from fluoropyrimidine-based regimens.^[23]^

Infectious causes demonstrated among the highest mortality risks, with septicemia exhibiting an SMR of 20.3 (95% CI: 13.88–28.66). Pneumonia and influenza showed an SMR of 88.69 (95% CI: 47.18–151.67), while other infectious and parasitic diseases demonstrated an SMR of 32.11 (95% CI: 18.69–51.41). These elevated infectious mortality risks reflect chemotherapy-induced immunosuppression, creating vulnerability to both community-acquired and opportunistic infections.^[24]^

Respiratory causes, particularly COPD, demonstrated an SMR of 17.57 (95% CI: 9.83–28.99), likely reflecting the high prevalence of smoking history in pancreatic cancer patients combined with chemotherapy-induced pulmonary toxicity.^[25]^ Metabolic complications, including diabetes mellitus, showed an SMR of 19.3 (95% CI: 11.78–29.81), potentially related to corticosteroid use and direct pancreatic dysfunction.

Notably, external causes revealed unexpected findings, with suicide and self-inflicted injury demonstrating an SMR of 113.68 (95% CI: 56.67–203.42). This finding underscores the profound psychological burden of pancreatic cancer diagnosis and highlights the critical need for integrated psychosocial support in comprehensive care models.^[26]^

### 3.9. Temporal patterns of non-cancer mortality risk

**Figure 5.**
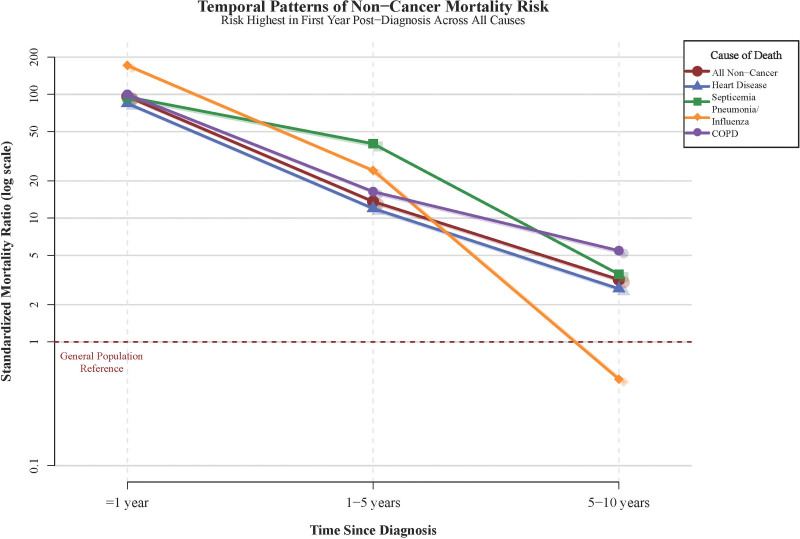
Temporal patterns of non-cancer mortality risk. Line plot illustrating the temporal evolution of standardized mortality ratios for major non-cancer causes by time since diagnosis (≤1 year, 1–5 years, 5–10 years). All causes demonstrate peak mortality risk in the first year (overall SMR 96.10) with subsequent decline. Infectious causes show most dramatic temporal concentration; cardiovascular causes show more gradual attenuation. COPD = chronic obstructive pulmonary disease, SMR = standardized mortality ratio.

Analysis of SMRs by time since diagnosis revealed a consistent pattern of highest risk in the immediate post-diagnosis period, with gradual attenuation over time (Fig. 5). Within the first year, overall non-cancer mortality demonstrated an SMR of 96.10 (95% CI: 85.25–107.95), declining to 13.63 (95% CI: 11.62–15.90) in years 1 to 5 and further to 3.19 (95% CI: 2.36–4.21) in years 5 to 10.

This temporal pattern was most pronounced for infectious causes, with septicemia showing an SMR of 95.41 (95% CI: 56.52–150.8) in the first year, decreasing to 39.93 (95% CI: 19.11–73.43) in years 1 to 5. Similarly, pneumonia and influenza demonstrated an SMR of 171.38 (95% CI: 85.43–306.66) within the first year, with no subsequent cases after 5 years, indicating that infectious mortality risk is predominantly concentrated in the acute treatment period.

Cardiovascular mortality risks followed similar temporal patterns but with more gradual attenuation. Heart disease demonstrated an SMR of 84.59 in the first year, declining to 11.98 in years 1 to 5 and 2.69 beyond 5 years. This pattern suggests both acute chemotherapy-related cardiotoxicity and longer-term cardiovascular consequences requiring extended surveillance and management.^[27,28]^

## 4. Discussion

This comprehensive population-based analysis of 9624 pancreatic cancer patients provides critical insights into the complex mortality landscape beyond cancer-specific deaths in the chemotherapy era. Our findings demonstrate that while pancreatic cancer-specific mortality dominates the overall mortality burden (90.6% of deaths), non-cancer mortality represents a clinically significant and potentially modifiable component of total mortality, particularly during the vulnerable first year following diagnosis.

The temporal concentration of non-cancer mortality risk in the immediate post-diagnosis period represents a crucial finding with direct clinical implications. Our analysis reveals that non-cancer mortality risk peaks dramatically within the first year (SMR 96.10), declining substantially in subsequent periods. This pattern suggests that the intersection of aggressive chemotherapy regimens, disease-related physiologic stress, and preexisting comorbidities creates a convergence of competing mortality risks that demands immediate attention and proactive management strategies.^[29]^ The magnitude of elevation for pneumonia/influenza (89-fold) and for heart disease exceeds that reported in most other cancers, likely reflecting both intensive regimen toxicity and compressed treatment timelines unique to pancreatic cancer.

Cardiovascular mortality emerged as the predominant non-cancer cause of death across all age groups and disease stages, with SMRs nearly higher than the general population. This finding aligns with emerging recognition of cardio-oncology as a critical discipline, particularly relevant to pancreatic cancer patients receiving cardiotoxic agents such as 5-fluorouracil, capecitabine, and oxaliplatin.^[30]^ The mechanisms underlying this elevated cardiovascular risk are multifactorial, encompassing direct cardiotoxic effects, including coronary vasospasm, endothelial dysfunction, and myocardial ischemia, combined with cancer-related systemic inflammation that accelerates atherosclerosis and increases thrombotic risk.^[31]^ The temporal pattern of cardiovascular mortality, with the highest risk in the first year, gradually attenuating over time, suggests both acute treatment-related toxicity and longer-term cardiovascular consequences requiring extended surveillance.

The substantial burden of infectious mortality in our cohort underscores the profound immunosuppressive effects of modern chemotherapy regimens. Septicemia demonstrated a 20-fold increased mortality risk compared with the general population, with pneumonia and influenza showing nearly elevation.^[32]^ These findings reflect the well-established neutropenic complications of intensive regimens like FOLFIRINOX, but also highlight the broader immunologic dysfunction associated with pancreatic cancer itself. The malnutrition, cachexia, and systemic inflammation characteristic of advanced pancreatic cancer create additional vulnerability to infectious complications, particularly in elderly patients with preexisting immune senescence.^[33]^

Our stage-stratified analysis reveals an unexpected paradox: early-stage patients show higher proportional non-cancer mortality (7.8% vs 5.1%) despite a better prognosis. This likely reflects survivor bias – early-stage patients live long enough for competing causes to manifest and may receive prolonged adjuvant therapy, accumulating cardiotoxic and immunosuppressive effects. The elevated cardiovascular SMR in stage I/II (12.4 vs 8.2) supports cumulative toxicity with extended exposure. Conversely, patients with liver metastases demonstrate distinct infectious vulnerability (septicemia SMR 24.7), suggesting hepatic metastatic burden compounds immunosuppression through impaired synthetic function and altered immune surveillance. These patterns inform risk stratification: early-stage survivors warrant long-term cardio-oncology surveillance, while liver metastasis patients may benefit from intensified antimicrobial prophylaxis during acute treatment.

Our age-stratified analysis reveals distinct mortality patterns that inform risk-stratified care approaches. Elderly patients (70–89 years) demonstrated the most compressed mortality timeline, with 66.4% of deaths occurring within the first year compared with 54.8% in younger patients (20–49 years). This pattern reflects not only the aggressive nature of pancreatic cancer in elderly populations but also the increased vulnerability to treatment toxicities in the setting of age-related organ dysfunction, polypharmacy, and frailty.^[34]^ The prominence of cardiovascular and respiratory causes in elderly patients suggests that comprehensive geriatric assessment and multidisciplinary care coordination may be essential for optimizing outcomes in this vulnerable population.

The unexpected finding of significantly elevated suicide mortality (SMR 113.68) illuminates the profound psychological burden associated with pancreatic cancer diagnosis and treatment.^[35]^ This finding, although based on a small absolute number of cases, represents an increased risk compared with the general population and highlights critical gaps in psychosocial support infrastructure. The devastating prognosis associated with pancreatic cancer, combined with debilitating symptoms, intensive treatment regimens, and financial toxicity, creates a complex psychological challenge requiring integrated mental health support from the time of diagnosis.

Our findings have immediate implications for clinical practice and healthcare system organization. The concentration of non-cancer mortality risk in the first year post-diagnosis suggests that this period requires intensive, multidisciplinary monitoring and intervention strategies. Cardio-oncology consultation should be considered for all patients receiving cardiotoxic regimens, with baseline cardiac assessment, serial monitoring during treatment, and long-term cardiovascular risk management.^[36]^ Similarly, infectious disease expertise may be valuable for managing the complex infectious complications that arise in this immunocompromised population.

The development of predictive models incorporating both cancer-specific and competing mortality risks could enhance shared decision-making regarding treatment intensity and supportive care measures.^[37]^ Our data suggest that certain patient subgroups, particularly elderly patients with multiple comorbidities, may benefit from modified treatment approaches that balance oncologic efficacy with competing mortality risks. This approach requires a sophisticated understanding of the relative contributions of cancer progression versus treatment toxicity to the overall mortality burden.

Future research priorities should include prospective studies examining the effectiveness of proactive cardiovascular and infectious disease management strategies in reducing non-cancer mortality.^[38]^ The integration of geriatric assessment tools, frailty indices, and comprehensive comorbidity evaluation into routine oncologic care may identify high-risk patients requiring modified treatment approaches or enhanced supportive care. Additionally, investigation of novel supportive care interventions, including cardioprotective strategies, antimicrobial prophylaxis protocols, and integrated psychosocial support programs, could potentially reduce the substantial non-cancer mortality burden we have identified.

### 4.1. Limitations

This study has several important limitations. SEER does not capture baseline comorbidity burden, specific chemotherapy regimens, immunotherapy/targeted therapy use, or dose modifications. While age stratification and population matching partially mitigate these limitations, individual-level adjustment is not possible. The absence of baseline cardiac status prevents distinguishing preexisting from treatment-induced cardiovascular disease, although the SMR magnitude (10-fold) substantially exceeds baseline prevalence, implicating treatment effects. Cause of death relies on death certificates and may be subject to misclassification when multiple conditions coexist. Our chemotherapy-treated cohort may not generalize to untreated patients. Future investigations incorporating comprehensive comorbidity data from linked electronic health records or Medicare claims will be essential for developing personalized risk assessment tools and targeted intervention strategies.

## 5. Conclusion

This comprehensive population-based analysis provides crucial evidence that non-cancer mortality represents a significant and potentially modifiable component of the total mortality burden in pancreatic cancer patients receiving chemotherapy. Our findings demonstrate that while pancreatic cancer-specific deaths dominate overall mortality (90.6%), the substantial elevation in cardiovascular and infectious mortality risks, particularly during the first year post-diagnosis, demands immediate clinical attention and systematic intervention strategies.

The identification of distinct temporal patterns, with higher non-cancer mortality in the first year declining substantially beyond 5 years, provides actionable insights for risk-stratified care delivery. Stage-stratified analysis reveals that early-stage survivors require long-term cardio-oncology surveillance for cumulative toxicity, while patients with liver metastases warrant intensified infection prevention during acute treatment. The prominence of cardiovascular and infectious complications underscores the urgent need for integrated cardio-oncology services and aggressive infection prevention protocols in comprehensive pancreatic cancer care.

These findings support the implementation of multidisciplinary care models incorporating baseline cardiovascular assessment, serial cardiac monitoring during treatment, and proactive management of infectious risks through enhanced supportive care measures. The elevated suicide mortality risk highlights critical gaps in psychosocial support infrastructure requiring systematic attention.

Future research examining the effectiveness of targeted interventions to reduce non-cancer mortality, including cardioprotective strategies, antimicrobial prophylaxis, and integrated mental health support, represents high-priority opportunities. The development of comprehensive risk prediction models incorporating both cancer-specific and competing mortality risks will enhance personalized treatment decision-making and optimize overall survival outcomes in this vulnerable patient population.

## Acknowledgments

We thank the Surveillance, Epidemiology, and End Results (SEER) program of the National Cancer Institute for providing access to the database used in this study. We also acknowledge the contributions of the participating SEER registries for their data collection efforts.

## Author contributions

**Conceptualization:** Zhirong Gao, Liye Bei, Kaili Peng.

**Data curation:** Zhirong Gao, Liye Bei, Kaili Peng.

**Formal analysis:** Zhirong Gao.

**Investigation:** Zhirong Gao, Liye Bei, Kaili Peng.

**Methodology:** Zhirong Gao, Liye Bei, Kaili Peng.

**Project administration:** Zhirong Gao, Liye Bei, Kaili Peng.

**Resources:** Zhirong Gao, Kaili Peng.

**Software:** Zhirong Gao, Liye Bei, Kaili Peng.

**Supervision:** Zhirong Gao, Liye Bei.

**Validation:** Zhirong Gao, Liye Bei, Kaili Peng.

**Visualization:** Zhirong Gao, Liye Bei, Kaili Peng.

**Writing – original draft:** Zhirong Gao.

**Writing – review & editing:** Zhirong Gao, Liye Bei, Kaili Peng.

## References

[1] Bray F Laversanne M Sung H. Global cancer statistics 2022: GLOBOCAN estimates of incidence and mortality worldwide for 36 cancers in 185 countries. CA Cancer J Clin. 2024;74:229–63. 38572751 10.3322/caac.21834

[2] Siegel RL Wagle NS Cercek A Smith RA Jemal A. Colorectal cancer statistics, 2023. CA Cancer J Clin. 2023;73:233–54. 36856579 10.3322/caac.21772

[3] Mizrahi JD Surana R Valle JW Shroff RT. Pancreatic cancer. Lancet. 2020;395:2008–20. 32593337 10.1016/S0140-6736(20)30974-0

[4] Burris HA Moore MJ Andersen J. Improvements in survival and clinical benefit with gemcitabine as first-line therapy for patients with advanced pancreas cancer: a randomized trial. J Clin Oncol. 1997;15:2403–13. 9196156 10.1200/JCO.1997.15.6.2403

[5] Conroy T Desseigne F Ychou M. FOLFIRINOX versus gemcitabine for metastatic pancreatic cancer. N Engl J Med. 2011;364:1817–25. 21561347 10.1056/NEJMoa1011923

[6] Von Hoff DD Ervin T Arena FP. Increased survival in pancreatic cancer with nab-paclitaxel plus gemcitabine. N Engl J Med. 2013;369:1691–703. 24131140 10.1056/NEJMoa1304369 PMC4631139

[7] Golan T Hammel P Reni M. Maintenance olaparib for germline BRCA-mutated metastatic pancreatic cancer. N Engl J Med. 2019;381:317–27. 31157963 10.1056/NEJMoa1903387 PMC6810605

[8] Rahib L Smith BD Aizenberg R Rosenzweig AB Fleshman JM Matrisian LM. Projecting cancer incidence and deaths to 2030: the unexpected burden of thyroid, liver, and pancreas cancers in the United States. Cancer Res. 2014;74:2913–21. 24840647 10.1158/0008-5472.CAN-14-0155

[9] Herrmann J. Adverse cardiac effects of cancer therapies: cardiotoxicity and arrhythmia. Nat Rev Cardiol. 2020;17:474–502. 32231332 10.1038/s41569-020-0348-1 PMC8782611

[10] Gourgou-Bourgade S Bascoul-Mollevi C Desseigne F. Impact of FOLFIRINOX compared with gemcitabine on quality of life in patients with metastatic pancreatic cancer: results from the PRODIGE 4/ACCORD 11 randomized trial. J Clin Oncol. 2013;31:23–9. 23213101 10.1200/JCO.2012.44.4869

[11] Freifeld AG Bow EJ Sepkowitz KA. Clinical practice guideline for the use of antimicrobial agents in neutropenic patients with cancer: 2010 update by the Infectious Diseases Society of America. Clin Infect Dis. 2011;52:e56–93. 21258094 10.1093/cid/cir073

[12] Zamorano JL Lancellotti P Rodriguez Muñoz D. 2016 ESC Position Paper on cancer treatments and cardiovascular toxicity developed under the auspices of the ESC Committee for Practice Guidelines: the Task Force for Cancer Treatments and Cardiovascular Toxicity of the European Society of Cardiology (ESC). Eur Heart J. 2016;37:2768–801. 27567406 10.1093/eurheartj/ehw211

[13] Koene RJ Prizment AE Blaes A Konety SH. Shared risk factors in cardiovascular disease and cancer. Circulation. 2016;133:1104–14. 26976915 10.1161/CIRCULATIONAHA.115.020406 PMC4800750

[14] Ellis L Woods LM Estève J Eloranta S Coleman MP Rachet B. Cancer incidence, survival and mortality: explaining the concepts. Int J Cancer. 2014;135:1774–82. 24945976 10.1002/ijc.28990

[15] Armenian SH Lacchetti C Barac A. Prevention and monitoring of cardiac dysfunction in survivors of adult cancers: American Society of Clinical Oncology clinical practice guideline. J Clin Oncol. 2017;35:893–911. 27918725 10.1200/JCO.2016.70.5400

[16] Bradley CJ Yabroff KR Dahman B Feuer EJ Mariotto A Brown ML. Productivity costs of cancer mortality in the United States: 2000–2020. JNCI. 2008;100:1763–70. 19066273 10.1093/jnci/djn384 PMC2720777

[17] Kleeff J Korc M Apte M. Pancreatic cancer. Nat Rev Dis Primers. 2016;2:16022. 27158978 10.1038/nrdp.2016.22

[18] McGuigan A Kelly P Turkington RC Jones C Coleman HG McCain RS. Pancreatic cancer: a review of clinical diagnosis, epidemiology, treatment and outcomes. World J Gastroenterol. 2018;24:4846–61. 30487695 10.3748/wjg.v24.i43.4846 PMC6250924

[19] Duggan MA Anderson WF Altekruse S Penberthy L Sherman ME. The Surveillance, Epidemiology, and End Results (SEER) program and pathology: toward strengthening the critical relationship. Am J Surg Pathol. 2016;40:e94–e102. 27740970 10.1097/PAS.0000000000000749 PMC5106320

[20] Friedman S Negoita S. History of the Surveillance, Epidemiology, and End Results (SEER) program. J Natl Cancer Inst Monogr. 2024;2024:105–9. 39102881 10.1093/jncimonographs/lgae033 PMC11300016

[21] Noone A-M Lund JL Mariotto A. Comparison of SEER treatment data with Medicare claims. Med Care. 2016;54:e55–64. 24638121 10.1097/MLR.0000000000000073 PMC4981219

[22] Noone A-M Mariotto AB Hong YD Enewold L. Assessing 1-year comorbidity prevalence and its survival implications in Medicare beneficiaries diagnosed with cancer: insights from a new SEER-Medicare resource. Cancer Epidemiol Biomarkers Prev. 2025;34:182–9. 39373617 10.1158/1055-9965.EPI-24-0833 PMC11717627

[23] Moslehi JJ. Cardiovascular toxic effects of targeted cancer therapies. N Engl J Med. 2016;375:1457–67. 27732808 10.1056/NEJMra1100265

[24] Kuderer NM Dale DC Crawford J Cosler LE Lyman GH. Mortality, morbidity, and cost associated with febrile neutropenia in adult cancer patients. Cancer. 2006;106:2258–66. 16575919 10.1002/cncr.21847

[25] Rawla P Sunkara T Gaduputi V. Epidemiology of pancreatic cancer: global trends, etiology and risk factors. World J Oncol. 2019;10:10–27. 30834048 10.14740/wjon1166 PMC6396775

[26] Curra M Gabriel AF Ferreira MBC. Incidence and risk factors for oral mucositis in pediatric patients receiving chemotherapy. Support Care Cancer. 2021;29:6243–51. 33846825 10.1007/s00520-021-06199-5

[27] Couch LS López-Fernández T Lyon AR. The ‘Ten Commandments’ for the 2022 European Society of Cardiology Guidelines on cardio-oncology. Eur Heart J. 2022;44:10–1. 10.1093/eurheartj/ehac666 36420674

[28] López-Fernández T Lyon AR Herrmann J. 2022 ESC Guidelines on cardio-oncology: how can we improve the cardiovascular health of patients with cancer and cancer survivors? Eur Heart J Cardiovasc Pharmacother. 2022;9:4–5. 36107817 10.1093/ehjcvp/pvac051 PMC9923207

[29] Templeton AJ McNamara MG Šeruga B. Prognostic role of neutrophil-to-lymphocyte ratio in solid tumors: a systematic review and meta-analysis. J Natl Cancer Inst. 2014;106:dju124. 24875653 10.1093/jnci/dju124

[30] de Boer RA Meijers WC van der Meer P van Veldhuisen DJ. Cancer and heart disease: associations and relations. Eur J Heart Fail. 2019;21:1515–25. 31321851 10.1002/ejhf.1539 PMC6988442

[31] Charo IF Taubman MB. Chemokines in the pathogenesis of vascular disease. Circ Res. 2004;95:858–66. 15514167 10.1161/01.RES.0000146672.10582.17

[32] Lyman GH Khorana AA Kuderer NM. Venous thromboembolism prophylaxis and treatment in patients with cancer: American Society of Clinical Oncology clinical practice guideline update. J Clin Oncol. 2013;31:2189–204. 23669224 10.1200/JCO.2013.49.1118

[33] Fearon K Strasser F Anker SD. Definition and classification of cancer cachexia: an international consensus. Lancet Oncol. 2011;12:489–95. 21296615 10.1016/S1470-2045(10)70218-7

[34] Tukenova M Guibout C Oberlin O. Role of cancer treatment in long-term overall and cardiovascular mortality after childhood cancer. J Clin Oncol. 2010;28:1308–15. 20142603 10.1200/JCO.2008.20.2267

[35] Ma Y Lyu J Yang B. Incidence and risk factors of suicide among patients with pancreatic cancer: a population-based analysis from 2000 to 2018. Front Oncol. 2022;12:972908. 36059612 10.3389/fonc.2022.972908 PMC9437642

[36] Cardinale D Colombo A Bacchiani G. Early detection of anthracycline cardiotoxicity and improvement with heart failure therapy. Circulation. 2015;131:1981–8. 25948538 10.1161/CIRCULATIONAHA.114.013777

[37] Gloeckler Ries LA Reichman ME Lewis DR Hankey BF Edwards BK. Cancer survival and incidence from the Surveillance, Epidemiology, and End Results (SEER) program. Oncologist. 2003;8:541–52. 14657533 10.1634/theoncologist.8-6-541

[38] Strobel O Neoptolemos J Jäger D Büchler MW. Optimizing the outcomes of pancreatic cancer surgery. Nat Rev Clin Oncol. 2019;16:11–26. 30341417 10.1038/s41571-018-0112-1

